# Formulation of *Blumea balsamifera*, *Anredera cordifolia*, and *Phyllanthus niruri* Extracts as Potential Anti-Inflammatory Agents

**DOI:** 10.3390/cimb48040405

**Published:** 2026-04-15

**Authors:** Siti Martinah Pajriah, Dyah Iswantini, Henny Purwaningsih, Banaz Jalil, Min Rahminiwati, Novriyandi Hanif

**Affiliations:** 1 Department of Chemistry, Faculty of Mathematics and Natural Sciences, IPB University, Bogor 16680, Indonesia; martinahsiti@apps.ipb.ac.id (S.M.P.); hennypu@apps.ipb.ac.id (H.P.); nhanif@apps.ipb.ac.id (N.H.); 2 Tropical Biopharmaca Research Center, IPB University, Bogor 16128, Indonesia; 3 Pharmacognosy and Phytotherapy Group, UCL School of Pharmacy, 29-39 Brunswick Square, London WC1N 1AX, UK; b.jalil@ucl.ac.uk; 4 School of Veterinary Medicine and Biomedical Sciences, IPB University, Bogor 16680, Indonesia; minrahminiwati@gmail.com

**Keywords:** *Anredera cordifolia*, *Blumea balsamifera*, COX-2, inflammation, *Phyllanthus niruri*

## Abstract

Inflammation is an immune response to foreign substances, pathogens, and cellular damage, characterized by redness, pain, and swelling. The use of synthetic anti-inflammatory drugs may cause adverse side effects, prompting the need for natural alternatives. This study aimed to evaluate the total flavonoid content and anti-inflammatory activity of single and combined extracts of sembung (*Blumea balsamifera*) [S], binahong (*Anredera cordifolia*) [B], and meniran (*Phyllanthus niruri*) [M] leaves. Extraction was performed using a 70% ethanol and water solution. Total flavonoid content was determined spectrophotometrically using quercetin as the standard. LC-HR-MS/MS results showed the presence of flavonoids, terpenoids, phenolics, and amino acids in each single extract (S, B, and M). Anti-inflammatory activity was assessed through the inhibition of albumin denaturation and the inhibition of cyclooxygenase-2 (COX-2). The highest flavonoid content (35.27 mg QE/g DW) in single extracts was found in B of the ethanolic extract, while the highest flavonoid content (40.24 mg QE/g DW) in all formulations was discovered in a combination of the ethanolic extract S:B:M (1:0:1). Moreover, the ethanolic extract S:B:M (1:0:1) gave the strongest inhibition (76.47%) of the albumin denaturation at 300 µg/mL and the strongest inhibition (98.5%) of the COX-2 inhibition at 100 µg/mL. Sembung extract was highest for reducing the expression of pro-inflammatory cytokines and TNF-α was 97.66% at an extract concentration of 15.625 ppm. As for S, S:B:M (1:0:1) and S:B:M (1:1:1) can reduce the expression of pro-inflammatory cytokines, as IL-6 was 100% in 0 pg/mL at an extract concentration of 15.625 ppm.

## 1. Introduction

Inflammation is the immune system’s biological response triggered by various factors, including foreign substances, pathogens, damaged cells, as well as toxic compounds entering the body. This response can be acute or chronic and may lead to serious diseases such as heart disease, lung disease, and rheumatoid arthritis [1]. Signs of inflammation include redness, heat, swelling, pain, and loss of function [2]. Many human diseases stem from the cyclooxygenase (COX) and lipoxygenase (LOX) pathways within the arachidonic acid (AA) pathway [3]. Eicosanoids like leukotrienes, prostaglandins, and thromboxanes are primary inflammatory mediators, requiring these enzymes for their physiological production [4]. During inflammation, markers such as reactive oxygen species (ROS), reactive nitrogen species (RNS), cytokines, and prostaglandins are present in macrophages, driving the inflammatory process [5].

Inflammation typically results from elevated prostaglandin production facilitated by the COX-2 enzyme, making COX-2 inhibition a common strategy in anti-inflammatory therapy [6,7]. COX is an enzyme that limits the rate of converting AA to prostaglandins and exists in two isoenzymes, COX-1 and COX-2 [8]. The COX-2 protein specifically catalyzes the formation of prostaglandins from arachidonic acid. Through a variety of mechanisms, including increased vasodilation, improved blood vessel permeability, and pain receptor sensitization, prostaglandins generated by COX-2 contribute to the inflammatory process [5]. COX-2 undergoes a significant increase due to stimulation from proinflammatory cytokines during inflammation [8].

The outer membrane of Gram-negative bacteria contains lipopolysaccharide (LPS), which activates the host’s immune response to inflammation. Consequently, the immune system increases the production of pro-inflammatory mediators, cytokines, as well as chemokines. Cytokines serve as key mediators in the inflammatory process. Pro-inflammatory cytokines, such as interleukin (IL)-6 and tumor necrosis factor alpha (TNF-α), are produced and released through pathways like nuclear factor kappa beta (NF-kβ) and mitogen-activated protein kinase (MAPK) [9]. TNF-α is considered a “master regulator” of pro-inflammatory cytokine production. It plays a crucial role in recruiting and activating inflammatory cells and is believed to be essential in many chronic inflammatory diseases’ development [10]. Besides its impact on hepatocytes and lymphocytes, IL-6 also exerts other effects commonly observed in chronic inflammatory conditions [11].

**Figure 1:**
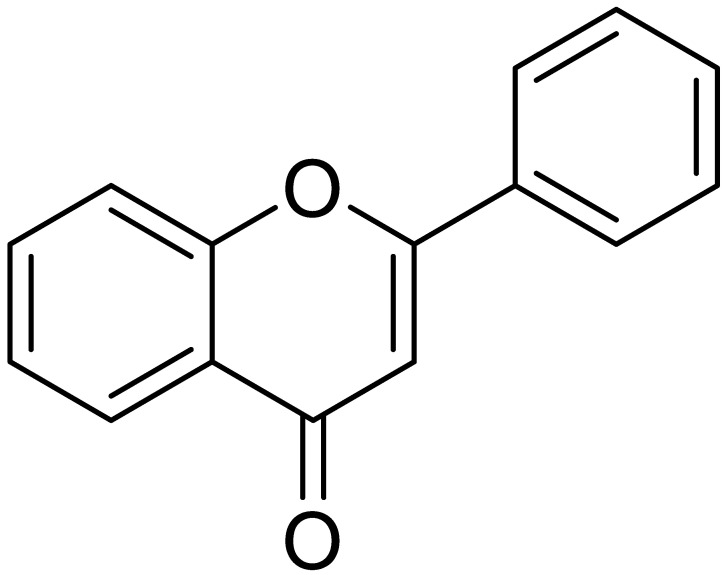
Structure of flavonoid.

Nonsteroidal anti-inflammatory drugs (NSAIDs) are commonly utilized to treat various inflammatory conditions. To minimize gastrointestinal side effects, selective COX-2 inhibitors were developed and introduced [3]. However, this application increases the risk of cardiovascular complications due to a decrease in prostaglandin I2 (PGI2) levels. Medical plants with potential anti-inflammatory activities, e.g., sembung (*Blumea balsamifera*) [12], binahong (*Anredera cordifolia*) [2], and meniran (*Phyllanthus niruri*) [13], could provide an alternative option. Such treatment efforts can utilize a combination of several plant extracts. Combining plant extracts into a single formula yields synergistic effects [2]. These medicinal plants contain bioactive metabolites such as flavonoids. Flavonoids, a type of phenolic compound, have potential pharmacological effects, including anti-inflammatory properties [14,15]. The *Indonesian Herbal Pharmacopoeia* (2017) reported that sembung leaves contain 1.31% flavonoids expressed as quercetin, binahong leaves have 1.74% flavonoids expressed as rutin, and meniran herbs contain 3.20% flavonoids expressed as quercetin [16]. Figure 1 shows the structure of a flavonoid.

Anti-inflammatory properties of sembung, binahong, and meniran have been reported in several studies. Maulydia et al. reported that the protein inhibition and denaturation levels of the *B. balsamifera* ethanolic extract were 103.85 ppm and 97.23 ppm, respectively. The findings demonstrate that the ethanolic extract of *B. balsamifera* is capable of reducing inflammation [14]. Binahong leaves (*Anredera cordifolia*) showed anti-inflammatory activity in a membrane stability assay using human red blood cells [17]. Wiratmini et al.’s study investigated the *P. niruri* leaf extract’s in vitro effects on pro-inflammatory cytokines using RAW 264.7 macrophage cells. In cells stimulated with LPS, treatment with the extract notably decreased the levels of TNF-α and IL-1β, with 50 µg/mL being the most effective dose. These findings suggest that *P. niruri* leaf extract has promising anti-inflammatory properties and deserves further exploration as a possible treatment for inflammatory diseases [5]. All three plants have been individually reported to exhibit anti-inflammatory activity. However, to the best of our knowledge, no study has investigated the combined effects of sembung, binahong, and meniran leaf extracts as anti-inflammatory agents [2]. According to Askar et al. (2025), the significance of traditional medicine has been highlighted by the introduction of polyherbal formulations that combine diverse medicinal plants with chemical compounds for treating diseases, and reports have documented various polyherbal formulations and their pharmacological activities [18]. This study was designed as an in vitro experimental investigation to explore the potential synergistic anti-inflammatory activity of a combination formulation of sembung, binahong, and meniran leaf extracts, which has not been previously reported. The anti-inflammatory activity was evaluated through protein denaturation inhibition, COX-2 enzyme inhibition, and proinflammatory cytokine suppression assays. It was hypothesized that the combined extracts would exhibit synergistic inhibitory effects compared to the individual extracts.

## 2. Materials and Methods

### 2.1. Preparation of the Sample

**Figure 2:**
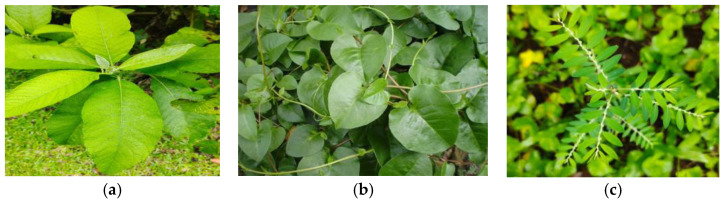
(**a**) Sembung leaf, (**b**) binahong leaf, (**c**) meniran leaf.

The samples were obtained from the Tropical Biopharmaceutical Study Center plantation at LPPM, IPB, and then examined at the Botanical Laboratory, National Research and Innovation Agency (BRIN), Cibinong, as shown in Figure 2. The collection numbers obtained from the Tropical Biopharmaca Research Center Database, LRI-PGKH IPB, are BMK0044052015 for *Blumea balsamifera*, BMK0155092016 for *Anredera cordifolia*, and BMK0194092016 for *Phyllanthus niruri*. In this study, 20 kg of fresh leaves of sembung, binahong, and meniran were collected. The leaves were initially rinsed under running water to remove dirt. They were then dried in the shade to avoid direct sunlight. Once dried, the leaves were ground utilizing an 80-mesh grinder and kept in clean, dry containers [19]. The codes for Sembung, Binahong, and Meniran are S, B, and M, respectively.

### 2.2. Extraction of the Sample

Extraction was performed using the maceration method [20] with distilled water and 70% ethanol solvents at room temperature. Powdered samples were prepared with distilled water and 70% ethanol solvents at room temperature. Sembung, binahong, and meniran leaf powders were weighed at a sample-to-solvent ratio of 1:10 (25 g in 250 mL). Before being filtered, the samples and solvents were shaken and allowed to stand for a full day. After being separated, the filtrate was repeated three times. A rotary evaporator operating at 40 °C was used to concentrate the filtrate. Weighing was done on the final extract. Equation (1) was used to calculate the yield.

(1) $$Yield\;(\%)=\frac{a}{b(1-c/100)}\times 100\%$$

Abbreviations: a = extract weight (g); b = sample weight (g); c = moisture content (%).

### 2.3. Phytochemical Analysis

Standard protocols were used for phytochemical testing [21]. Three reagents were utilized for alkaloids: Bouchardat (a brown to black precipitate shows alkaloids), Dragendorff (a red precipitate indicates alkaloids), and Mayer (10 drops of test solution with 2 drops of reagent; a white precipitate indicates alkaloids). Ten drops of the test solution were mixed with 0.1 g of magnesium powder, 1 mL of concentrated HCl, and 2 mL of amyl alcohol to perform the flavonoid test. The presence of flavonoids was identified by yellow, orange, or red hues in the amyl alcohol layer. For saponins, mix 5 drops of hot water with 10 drops of the test solution, then shake to create foam. The presence of saponins is indicated by the froth that remains after adding one drop of 2N iodide acid. To test for tannin, boil 0.1 g of the sample extract with 5 mL of distilled water, filter the liquid, and then add 3 drops of FeCl_3_. Tannin is indicated by a dark blue or greenish-black hue.

### 2.4. Determination of Total Flavonoid Content

The method from the second edition of the *Indonesian Herbal Pharmacopoeia* was used to calculate the total flavonoid content (Ministry of Health of the Republic of Indonesia, 2017) [16]. After dissolving 0.2 g of the extract in 25 mL of ethanol, it was agitated for half an hour. It was then adjusted with ethanol after being filtered into a 25 mL volumetric flask. Quercetin was diluted to six concentrations (0.5, 1.0, 2.0, 3.0, 4.0, and 5.0 mg/L) in ethanol to create a calibration curve. Then, 1.5 mL of ethanol, 0.1 mL of 10% AlCl_3_, 0.1 mL of 1 M sodium acetate, and 2.8 mL of water were combined with 0.5 mL of the sample extract or standard solution.

After homogenizing the mixture with a vortex, it was left standing at room temperature for half an hour. A UV-Vis spectrophotometer (Shimadzu UV-1780, Kyoto, Japan) set to 425 nm was used for the measurements. The total flavonoid concentration of each extract was measured three times and expressed in mg quercetin equivalent (QE)/g DW. Equation (2) forms the basis for the formula.

(2) $$C=\frac{C_{q}\times V}{m}\times FP$$

Abbreviations:

C = Total flavonoid (mg QE/g DW).

C_q_ = Concentration of sample (mg/L).

V = Volume of extract (L).

M = Weight of extract (g).

FP = Dilution factor.

### 2.5. Preparation of Combination Extracts of S, B, and M

Combination extracts of S, B, and M were prepared at a final concentration of 300 ppm in 7 samples. The formulation details are presented in Table 1. This formulation aimed to evaluate the potential individual and synergistic effects. According to Askar et al. [18], the traditional medicine’s significance has been highlighted by the introduction of polyherbal formulations that combine diverse medicinal plants with chemical compounds beneficial for treating diseases.

### 2.6. The Inhibition of Albumin Denaturation

A test for anti-inflammatory activity was conducted via the albumin denaturation inhibition method [22]. Each sample was added to 5 mL of a 0.15% BSA solution in tris-buffered saline (TBS) at a concentration of 300 ppm, and the mixture was incubated for 30 min at 25 °C. After heating the combination to 70 °C for five minutes, it was cooled by submerging it in water for around ten minutes. Following cooling, a vortex mixer was used to homogenize the mixture, and a UV-Vis spectrophotometer set to 660 nm was used to measure the absorbance. Three iterations of the test were conducted.

### 2.7. The Inhibition of COX-2 Activity and IC50

The SBM leaf extracts’ formulations with the highest anti-inflammatory activity in the albumin denaturation inhibition test were then tested for inhibition of COX-2 activity according to Tuwalaid et al. [23]. The test method followed the kit protocol, which included the COX-2 reaction procedure, COX-2 pre-reaction preparation, and the COX-2 reaction process. The next stage involved ELISA buffer preparation, special test pre-reaction preparation, COX-2 reaction dilution, application to 96-well plates, and measurement using an Elisa microplate reader nanospectrophotometer (Biotech, Winooski, VT, USA) at λ = 405–420 nm.

The IC_50_ value was determined by assessing the inhibitory activity of the sample over a range of concentrations selected to yield inhibition levels both above and below 50%. A dose–response curve was generated by plotting the logarithm of the concentrations against the percentage inhibition, and the IC_50_ value was derived using nonlinear regression analysis.

### 2.8. Determination of Pro-Inflammatory Cytokines

TNF-α and IL-6 concentrations in RAW 264.7 were used to quantify cytokine inhibitory activity [24]. The Primate Research Center, IPB University, Bogor, Indonesia, provided RAW 264.7 macrophage cells. The ELISA approach was used to quantify macrophages produced by lipopolysaccharide. A 96-well plate was seeded with 1 × 10^3^ RAW 264.7 cells per well and cultured for 24 h. Test samples were added at concentrations of 7.8125 µg/mL, 15.625 µg/mL, and 31.25 µg/mL after the medium was changed with new medium. The cells were once more cultured for two hours. The cells were then incubated for a further 24 h after being stimulated with 1 µg/mL lipopolysaccharide. After gathering the medium, it was centrifuged for ten minutes at 2000 rpm. TNF-α and IL-6 levels were measured in the supernatant using the Elabscience mouse ELISA kit in accordance with the usage instructions. Equation (3) was used to calculate the inhibition of pro-inflammatory cytokines.

(3) $$\%\;inhibition=\left[\frac{{Concentration}_{negative\;control}{-Concentration}_{sample}}{{Concentration}_{negative\;control}}\right]\times 100\%$$

### 2.9. Testing of Bioactive Metabolites in Sembung Leaves, Binahong Leaves, and Meniran Using the LC-HRMS/MS Method

LC-HRMS/MS was used to tentatively identify a single extract [20]. After dissolving 5 mg of extract in 1 mL of methanol, the mixture was sonicated for 30 min. The mixture was then filtered through a 0.22 μm PTFE membrane. Metabolites were separated and identified using UHPLC Vanquish Tandem Q Exactive Plus Orbitrap HRMS from ThermoScientific, Waltham, MA, USA. The column was an Accucore C18 [100 × 2.1 mm, 1.5 μm (ThermoScientific)]. The mobile phase consisted of 0.1% formic acid in water (A) and 0.1% formic acid in acetonitrile (B). The gradient elution was set as follows: 15%, 15–55%, 55–95%, 95%, and 15% (B) at 0–1, 1–20, 20–23, 23–28, and 28–30 min, respectively. The flow rate was set at 0.2 mL/min, with an injection volume of 2.0 μL. The ionization source employed ESI in both positive and negative modes within the *m*/*z* range of 100–1500. The capillary temperature was maintained at 320 °C, the spray voltage was 3.8 kV, and the sheath and auxiliary gas flows were 15 and 3 mL/min, respectively. The automatic gain control (AGC) was set to 3 × 10^6^, and the injection time was set to 100 ms. Full MS/dd MS2 and full-scan data capture with a resolution of 70,000 FWHM were the scan types utilized. Additionally, 18, 35, and 53 eV were the collision energies that were employed. Data from HRMS UHPLC-Q-Orbitrap was processed using Compound Discoverer 2.2 with a database constructed from metabolite data on *B. balsamifera*, *A. cordifolia*, *P. niruri* L.

### 2.10. Data Analysis

The COX-2 activity inhibition test results for the optimal extract formulation were analyzed using one-way ANOVA with IBM SPSS Statistics 21 [25] to identify any significant differences. Additionally, the secondary metabolites of S, B, and M were characterized using an LC-MS/HRMS spectrometer.

## 3. Results

### 3.1. The Extraction Yield

The S, B, and M extraction was performed using water and 70% ethanol solvents. Table 2 shows that ethanol yielded the highest results for all samples, with B extract being the highest at 78.32%, indicating a higher concentration of dissolved compounds compared to S and M.

### 3.2. Phytochemical Screening Results

Table 3 displays the sample extract’s phytochemical test results.

### 3.3. Total Flavonoid Content

**Figure 3:**
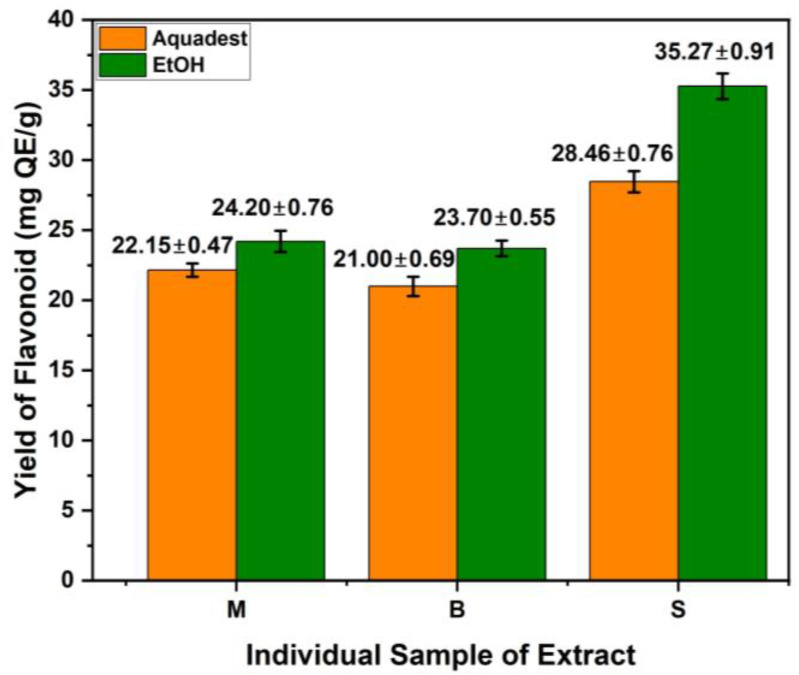
Average total flavonoid content of each individual extract of S, B, and M. Values are expressed as mean ± SD (n = 3).

Figure 3 presents the total flavonoid testing results for each individual extract. The ethanol extract of S yielded the highest total flavonoid content at 35.27 mg QE/g DW, while the water extract of B had the lowest at 21.00 mg QE/g DW. Overall, the ethanol extracts from the three plants had higher total flavonoid contents than their water extracts.

**Figure 4:**
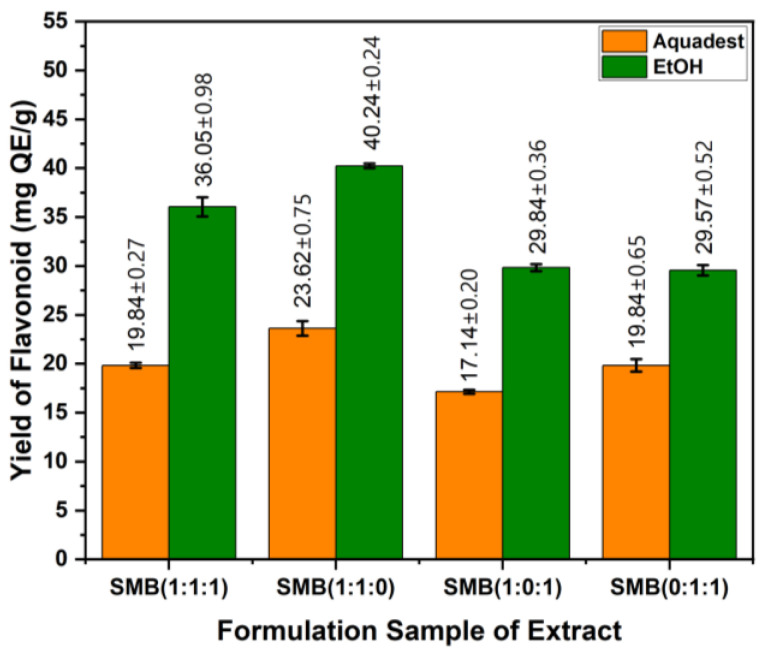
Average total flavonoid content of each formulation extract of S, M, and B. Values are expressed as mean ± SD (n = 3).

For the extract formulation, the highest total flavonoid content (40.24 mg QE/g DW) was obtained with EtOH from S:B:M (1:0:1), while the lowest (17.14 mg QE/g DW) was obtained with water from S:B:M (1:1:0) (Figure 4). Overall, the 70% EtOH extract formulation showed higher total flavonoid levels than the water extract formulation. This indicates that 70% EtOH is a more effective solvent for extracting flavonoid compounds than water.

### 3.4. The Inhibition of Albumin Denaturation

**Figure 5:**
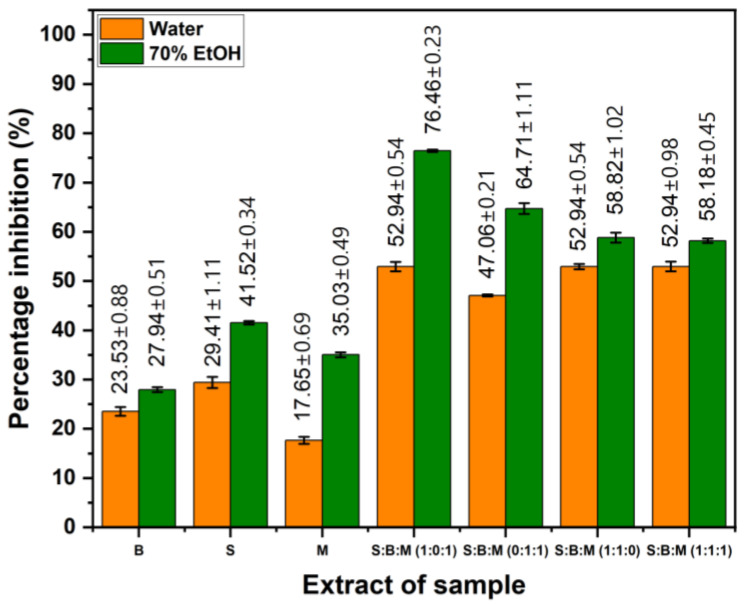
The percentage value of albumin denaturation inhibition of every single extract and formulation S, M, and B. Values are expressed as mean ± SD (n = 3).

The anti-inflammatory test results, obtained using the albumin denaturation method, are presented in Figure 5. The 70% ethanol extract of S exhibited the greatest inhibition level at 41.81% among all individual extracts. On the other hand, the combination extract, specifically the S:B:M (1:0:1) formulation extracted with ethanol, produced the greatest overall inhibition value, with an inhibition percentage of 76.47%. For both single extracts and combination formulations, extracts prepared with ethanol generally demonstrated greater protection against albumin denaturation than those prepared with distilled water.

The albumin denaturation inhibition values of the 70% ethanol extracts were higher than those of the water extracts. Therefore, statistical analysis was focused on the ethanol extracts to provide a more relevant evaluation of anti-inflammatory activity. Table 4 displays the findings of the statistical analysis of the percentage of albumin denaturation inhibition. All samples had statistically significant differences from one another at a 95% confidence level (*p* < 0.05), according to Tukey’s post hoc test. These significant differences are indicated by different codes for each sample, indicating that each treatment produced a distinctly different percentage of albumin denaturation inhibition.

### 3.5. Anti-Inflammatory Activity Based on COX-2 Inhibition Value

**Figure 6:**
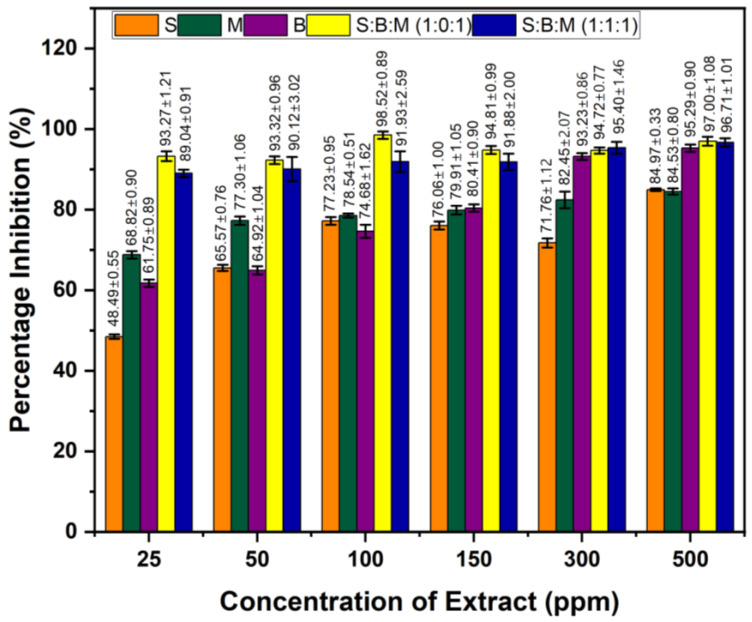
COX-2 inhibition values of 70% ethanol extracts of S and formulation S, M, and B. The values 25, 50, 100, 150, 300, and 500 are sample concentrations in µg/mL. Values are expressed as mean ± SD (n = 3).

Based on the albumin denaturation inhibition test results, the extracts progressing to COX-2 testing included the 70% ethanol extracts of S, M, and B, along with the 70% ethanol formulations of S:B:M (1:0:1) and S:B:M (1:1:1). All samples were assessed at concentrations of 25, 50, 100, 150, 300, and 500 µg/mL. As shown in Figure 6, the single extracts exhibited inhibition percentages of 84.97%, 84.53%, and 95.29% at 500 µg/mL. For the extract combinations, the highest inhibition was 98.52% and 91.93% at 100 µg/mL. Sodium diclofenac was used as the positive control.

The COX-2 inhibition test demonstrated that all extracts showed strong inhibitory activity between 25 and 500 mg/L, with inhibition rates over 48% even at the lowest concentration. Consequently, all samples’ IC_50_ values were below 25 mg/L. The S:B:M (1:0:1) combination extract showed the highest potential with 92–98% inhibition at all concentrations and a very low estimated IC_50_ < 25 mg/L. A similar pattern was observed in the S:B:M (1:1:1) combination with inhibition values of 89–96% and an IC_50_ < 25 mg/L. Both combinations exhibited synergistic effects among secondary metabolites (flavonoids, terpenoids, and saponins), resulting in higher enzyme inhibition than the single extracts as shown in Table 5.

Single extracts M and B showed higher COX-2 inhibitory activity than S, with inhibition values exceeding 60% at 25 mg/L; therefore, the IC_50_ value was estimated to be below 25 mg/L. B showed a sharper increase in inhibition at medium concentrations (150–300 mg/L), while M exhibited stable activity across all concentrations; this aligns with its content of flavonoids, saponins, lignans, and tannins, which are known to inhibit the COX-2 pathway via the NF-κB mechanism and modulate inflammatory mediators. Extract S showed the lowest activity at 25 mg/L concentration, with 48.48% activity, indicating a lower potential compared to other single extracts. These results correlate with previous reports on the inhibition of denaturation albumin activity of single extracts S, B, and M in Figure 5. Additionally, the formulated extract exhibited a synergistic effect, resulting in higher inhibition values. Since all samples yielded inhibition values greater than 48% at 25 mg/L concentration, the IC_50_ values of all extracts were below 25 mg/L. Based on the data, IC_50_ values in individual and formulation extracts in Table 5.

The statistical analysis results of the COX-2 enzyme inhibition percentage are presented in Table 6. The analysis shows that the factors of concentration, type of treatment, and the interaction between the two have a significant effect on the COX-2 inhibition response at a 95% level (*p* < 0.05). Tukey’s post hoc test noted that the treatments in samples SM and SMB produced the highest inhibition percentages and were statistically different from the other treatments, as indicated by the distinct codes assigned to each treatment.

### 3.6. Identification of the Secondary Metabolites in the Samples

The LC-HR-MS/MS results are reported for the three extracts: S, B, and M. The secondary metabolite profiles of the individual extracts of S, B, and M are presented in Table 7. According to LC-HR-MS/MS analysis, the extract S contains flavonoids, phenols, and terpenoids. The extract B has flavonoids. The extract M has flavonoid and phenol groups.

The chemical structure of the secondary metabolite in extracts S, B, and M are flavonoid groups is shown in Table 8.

### 3.7. Inhibition of Single and Combination Extracts of the Samples

Inhibition testing was performed separately using single extracts and combination extracts from sembung leaves, as well as S:B:M (1:0:1), and S:B:M (1:1:1) extracts using EtOH solvent. The positive controls were BL and tetrahydropyrimidine, and the negative control was LPS. Immune cells that release proinflammatory cytokines including interleukin (IL)-6 and tumor necrosis alpha (TNF-α) were used in this test.

**Figure 7:**
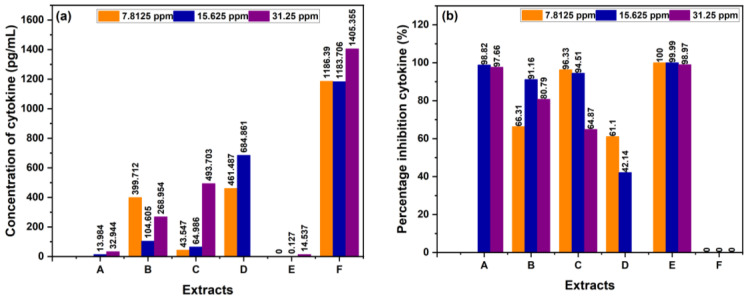
(**a**) Concentration cytokine of TNF-α, (**b**) % cytokine inhibition of TNF-α.

Table 9 shows that the sembung extract at 15.625 ppm had the lowest TNF-α concentration (13.984 pg/mL) and the highest inhibition percentage (97.66%). The S:B:M (1:0:1) combination extract at a concentration of 15.625 ppm had the lowest TNF-α concentration (104.605 pg/mL) and the highest inhibition percentage (91.16%). At 7.8125 ppm, the S:B:M (1:1:1) combination extract had the highest inhibition percentage (96.33%) and the lowest TNF-α concentration (43.547 pg/mL). The data above shows that the combination extract has a higher inhibition value than the single extract. In this TNF-α measurement, the single and combination extracts showed anti-inflammatory activity, as displayed in Figure 7.

**Figure 8:**
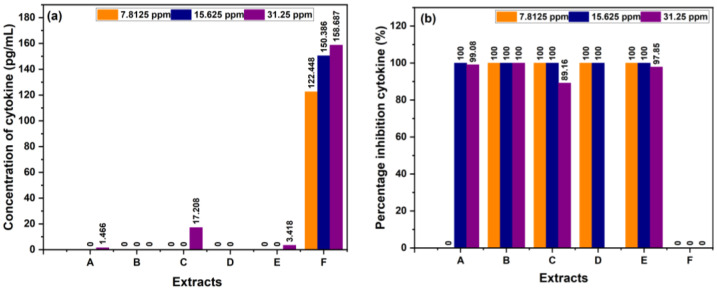
(**a**) Concentration cytokine of IL-6, (**b**) % cytokine inhibition of IL-6.

Table 10 shows that the single sembung extract at a concentration of 15.625 ppm had the lowest IL-6 concentration of 0 pg/mL with an inhibition value of 100%. The S:B:M (1:0:1) combination extract at all concentrations had an IL-6 concentration of 0 pg/mL with an inhibition value of 100%. Meanwhile, the S:B:M (1:1:1) combination extract at concentrations of 7.8125 ppm and 15.625 ppm had an IL-6 concentration of 0 pg/mL with an inhibition value of 100%. The data above shows that the combination extract has a higher inhibition value than the single extract. In this IL-6 measurement, the single and combination extracts showed anti-inflammatory activity, as presented in Figure 8.

Table 11 shows that single extracts and combination extracts exhibit higher anti-inflammatory activity against IL-6 than against TNF-α.

## 4. Discussion

All three extracts demonstrated that 70% ethanol was more effective than water, as indicated by extract B, which produced the highest yield of 78.32%, compared to extracts S and M. The most effective solvent was 70% ethanol, which can extract several bioactive metabolites [37]. The phytochemical test in these experiments, as previously reported, showed that the ethanolic extract of B contains alkaloids, flavonoids, and saponins, whereas the water extract does not have steroids or triterpenoids [2]. This variation is impacted by the plants’ growing locations, which can alter the secondary metabolites’ levels [38]. The ethanolic (70%) extract of S contains flavonoids, phenolics, and steroids/triterpenoids, while water extracts contained almost all compounds except alkaloids. Based on the previous reports, the ethanolic extract of S contains saponins that were not detected in this study [39]. The extract of M contains alkaloids, flavonoids, phenolics/tannins, and saponins, although this differs from the study reported, which also found steroids/triterpenoids [5]. Overall, extract M shows the most consistent bioactive metabolite content across both solvents.

The higher flavonoid content in ethanol extracts compared to water extracts may be due to the polarity of ethanol, which allows it to dissolve flavonoids more effectively. The literature reports that solvent characteristics during the extraction process play a crucial role in flavonoid stability and recovery, with ethanol being preferred for extracting semi-polar phenolic compounds such as flavonoids [40]. Many Indonesian herbal plants produce prominent flavonoids when ethanol is used as the extraction solvent [41]. Other research has also used ethanol extract in pharmacological evaluations, including laxative activity testing. Another study used ethanol extract to measure the laxative effect [42]. Based on the total flavonoid test results from the three plants, as reported by the *Indonesian Herbal Pharmacopoeia* [16], these results endorse using ethanol as an extraction solvent to increase flavonoid levels, which are linked to the extracts’ anti-inflammatory properties through mechanisms like protein stabilization and the inhibition of inflammatory enzymes. According to a study, flavonoids have potential as anti-inflammatory agents [15].

The highest flavonoid levels were found in the 70% EtOH formulation. Ethanol is an effective solvent because its moderate polarity allows it to dissolve polar compounds, extract phenolics [40], and better penetrate plant cell walls and membranes. Moreover, the solvent’s polarity affects both extraction efficiency and the bioactive metabolites acquired. According to studies, cold maceration effectively extracts thermolabile compounds like polyphenols by avoiding heat damage [43]. However, its effectiveness can be limited by the solvent’s ability to penetrate plant tissues and the duration of extraction [44]. Additionally, research shows that ethanolic extracts can inhibit COX-2 activity [45].

The single extract with the highest inhibition percentage was the 70% ethanol extract of S, at 41.81%. In contrast, the highest value was observed in the combination formulation, specifically the S:B:M (1:0:1) ethanol extract at 76.47%. The higher inhibition value compared to the study, where a single meniran extract had an inhibition percentage of 31.4%, suggests the possibility of a synergistic effect from the combination of extracts [2]. Overall, ethanol extracts showed greater inhibition of albumin denaturation than distilled water, both in single and combined extracts, with the highest value observed in the ethanol combination. This is thought to be because ethanol is more effective at extracting bioactive metabolites like flavonoids, terpenoids, and saponins that contribute to anti-inflammatory activity. The combination of several extracts showed the greatest effect, suggesting the possibility of synergism among metabolites from each plant. In other words, the ethanol extract combination showed the greatest potential and was selected for further testing against the COX-2 enzyme.

The secondary metabolite profiles of the individual extracts of S, B, and M are presented in Table 7. The LC-MS results showed that single extracts from S, B, and M contained flavonoids, terpenoids, phenolics, and amino acids. According to GC-MS, LC-HRMS, and UPLC-Q-Orbitrap HRMS analyses, sembung extract is rich in flavonoids, phenolic acids, alkaloids, terpenoids, as well as fatty acyls. Several studies have confirmed its anti-inflammatory activity [14,27,46]. According to previously reported research, binahong extract contains isoflavone aglycones, such as glycitein, and flavonoid compounds, including vitexin and kaempferol-3-O-β-D-galactoside-7-O-α-L-rhamnoside. Through protein stabilization and inflammatory pathway modification, several substances have been connected to anti-inflammatory effects [47]. Furthermore, LC-MS-based metabolomic analyses indicate that meniran extract contains flavonoids, tannins, and terpenoids, including epicatechin, quercetin, vitexin, rutin, kaempferol, astragalin, and apigenin [5,32].

According to studies, amino acids such as glutamine and arginine may help reduce inflammation, oxidative stress, along with proinflammatory cytokine levels in inflammatory bowel disease (IBD) [48]. Another study reported that histidine in IBD models inhibited NF-kB activation along with reduced TNF-α, IL-6, and other proinflammatory factors [49]. Phenolics and flavonoids, including quercetin, kaempferol, isorhamnetin, luteolin, and apigenin, have benefits, including anti-inflammatory activity. In the LC-MS data in Table 8, the flavonoid compounds found were kaempferol, retusin, isorhamnetin, tricin, skrofulein, hispidulin, apiin, rutin, and isoquercetin. It is believed that these compounds contribute to anti-inflammatory activity, as supported by the highest flavonoid levels observed in the single sembung extract and the S:B:M (1:0:1) and S:B:M (1:1:1) combination extracts, which respectively correlate with the % albumin denaturation inhibition value. Additionally, the statistical test results in Table 5 show that the single sembung extract and the S:B:M (1:0:1) and S:B:M (1:1:1) extract combinations produced the highest inhibition values against albumin denaturation.

Besides testing for COX-2 inhibition, the extract was evaluated for its effects on TNF-α and IL-6, which are key proinflammatory mediators. Inflammation is a vital biological response to injury and plays a role in the development of chronic diseases such as rheumatoid arthritis, IBD, and cancer [2]. Macrophages are central to this process, as exposure to lipopolysaccharides (LPS) from Gram-negative bacteria cell walls activates the immune system. This activation causes macrophages to release inflammatory markers including ROS, mediators like prostaglandin (PGE2) and nitric oxide (NO), and pro-inflammatory cytokines like TNF-α and IL-6. If not controlled, this response can lead to persistent tissue-damaging inflammation. Therefore, inhibiting LPS-induced macrophage activation is a key therapeutic target for inflammatory diseases [5,48].

The TNF-α and IL-6 assays explained that the combined extract had higher inhibition rates than the individual extracts, as detailed in Table 9 and Table 10. Furthermore, the overall extract proved its capacity to lower the expression of pro-inflammatory cytokines like TNF-α and IL-6, supported by these inhibition results. Past studies have indicated that many flavonoids reduce the expression of pro-inflammatory cytokines such as IL-6, IL-8, TNF-α, IL-1β, and monocyte chemotactic protein-1 (MCP-1) in RAW macrophages, peripheral blood mononuclear cells, and Jurkat T cells [50,51]. The findings suggest that flavonoids in red betel leaf extract may inhibit TNF-alpha expression by blocking nuclear factor kappa B (NF-κB), a transcription factor that regulates TNF-alpha production [52]. Moreover, the activation of NF-κB can increase COX-2 expression [5]. As an enzyme involved in inflammation, COX-2 catalyzes the formation of prostaglandins from arachidonic acid, which, by promoting vasodilation, increasing blood vessel permeability, and sensitizing pain receptors, plays a key role in the inflammatory process [53].

The body’s reaction to invading chemicals is inflammation. Through a variety of cell receptors, immune cells identify a variety of foreign substances, including bacteria, viruses, parasites, antigens, and toxins. Once identified, various proinflammatory pathways activate, leading to cytokine production and immune cell engagement, such as lymphocytes and macrophages, to eliminate foreign substances. If the body cannot resolve these substances early, the inflammatory response may escalate into a chronic phase, characterized by excess cytokines, chemokines, and inflammatory enzymes. Multiple receptor-mediated pathways, such as toll-like receptors (TLRs), mitogen-activated protein kinase (MAPK) pathways, and nuclear factor kappa-light-chain-enhancer of activated B cells (NF-κB), regulate over 50 inflammation-related genes [54]. Endothelial cells are stimulated by COX-2 and other cytokines, controlled by NF-κB, triggering signaling that attracts neutrophils. These neutrophils release prostaglandin E2 (PGE2) through COX-1 or COX-2 activity and produce cytokines, ROS, and histamine, all contributing to inflammation and pain [55,56]. Disruption of these pathways can lead to vascular proliferation, tissue damage, fibrosis, and secondary diseases such as arthritis, atherosclerosis, cardiovascular issues, Alzheimer’s, asthma, and cancer [57]. Flavonoids with anti-inflammatory effects are known to interact with multiple molecules in these pathways, decreasing the activity of inflammatory enzymes, chemokines, as well as cytokines.

## 5. Conclusions

The combination of sembung, binahong, and meniran leaf extracts demonstrated significant anti-inflammatory activity in vitro. The S:B:M (1:0:1) formulation showed the strongest effects, as evidenced by the high inhibition of protein denaturation and COX-2 activity, along with the significant suppression of pro-inflammatory cytokines (TNF-α and IL-6). These effects may be attributed to the presence of bioactive compounds, particularly flavonoids identified through LC-HR-MS/MS analysis.

Overall, the findings indicate that the combined extracts exhibit enhanced, potentially synergistic anti-inflammatory activity compared to individual extracts, highlighting their potential as a promising polyherbal anti-inflammatory formulation. Further studies, including in vivo evaluations, are recommended to confirm these findings.

## Figures and Tables

**Table 1:** The formulation sample of the extract.

Name of the Sample	Sample of the Extract	Formulation
1	S:B:M	1:0:0
2		0:1:0
3		0:0:1
4		1:0:1
5		1:1:0
6		0:1:1
7		1:1:1

**Table 2:** The extract yield of sembung, binahong, and meniran leaves.

Sample	Solvent	Concentrated Extract Weight (g)	Yield (%)
S	Water	7.05	35.23 ± 0.35
	70% EtOH	8.25	41.24 ± 0.86
B	Water	12.75	63.73 ± 0.38
	70% EtOH	15.66	78.32 ± 1.06
M	Water	6.92	34.58 ± 0.44
	70% EtOH	7.88	39.40 ± 0.28

**Table 3:** Phytochemical analysis of meniran, binahong, and sembung leaf extract.

Sample	Solvent	Phytochemical Test				
		Alkaloid	Flavonoid	Phenolic/Tannin	Steroid/Triterpenoid	Saponin
S	Water	-	+	+	+	+
	70% EtOH	-	+	+	+	-
B	Water	+	+	+	-	+
	70% EtOH	+	+	-	-	+
M	Water	+	+	+	-	+
	70% EtOH	+	+	+	-	+

Description: (+) contains secondary metabolite compounds; (-) contains no secondary metabolite compounds.

**Table 4:** Statistical analysis of denaturation albumin inhibition.

Sample	Percentage Inhibition of Denaturation Albumin (%)
B	27.94 ± 0.51 ^a^
M	35.03 ± 0.49 ^b^
S	41.52 ± 0.34 ^c^
S:B:M (1:1:1)	58.18 ± 0.45 ^d^
S:B:M (1:0:1)	76.46 ± 0.23 ^e^

Note: Values are expressed as mean ± SD (n = 3). Different superscript letters indicate statistically significant differences (*p* < 0.05) based on Tukey’s post hoc test.

**Table 5:** The IC_50_ values in individual and formulation extracts.

Sample	IC_50_ Approximation (mg/L)
S:B:M (1:0:1)	<25
S:B:M (1:1:1)	<25
S	28.04 ± 0.42
M	<25
B	<25

Note. Values are expressed as mean ± SD (n = 3).

**Table 6:** Statistical analysis of COX-2 inhibition.

Sample	Percentage Inhibition of COX-2 (%)
BL25	55.26 ± 0.87 ^a^
S50	70.67 ± 0.74 ^b^
B500	78.37 ± 1.49 ^c^
M100	78.59 ± 1.41 ^d^
S:B:M (1:1:1)150	92.51 ± 0.77 ^e^
S:B:M (1:0:1)300	95.11 ± 1.94 ^f^

Note: BL is BioLuric. Values are expressed as mean ± SD (n = 3). Different superscript letters indicate statistically significant differences (*p* < 0.05) based on Tukey’s post hoc test.

**Table 7:** Identification of the secondary metabolites and compounds in extract S, B, and M.

Sample	Compound	Formulation	Mass	RT [min]	Score	Classification	Ref.
S	Kaempferol	C_15_H_10_O_6_	286.048	11.409	94.8	Flavonoids	
	Retusin	C_19_H_18_O_7_	358.105	18.621	85.1	Flavonoids	
	Isorhamnetin	C_16_H_12_O_7_	316.058	13.171	85.5	Flavonoids	[26,27]
	Diisononyl phthalate	C_26_H_24_O_4_	418.308	27.051	97	Phthalic acids	
	Tricin (5,7,4′-trihydroxy-3′,5′-dimethoxyflavone)	C_17_H_14_O_4_	330.074	13.513	84	Flavonoids	[28]
	Choline	C_5_H_13_NO	103.1	1.077	94.7	Cholines	
	(+/-)-Usnic acid	C_18_H_16_O_7_	344.089	16.722	92.6	Dibenzorufan	
	Skrofulein	C_17_H_14_O_6_	314.079	16.072	88.2	Flavonoids	
	Santene	C_9_H_14_	122.11	14.216	91.6	Parenthydrocarbon	
	Cynarine	C_25_H_24_O_12_	516.126	9.636	96.5	Polyphenol	
	Hispidulin	C_16_H_12_O_6_	300.063	12.918	92.4	Flavonoids	[29]
	Chlorogenic acid	C_16_H_18_O_9_	354.095	6.09	98.9	Phenols	
	Gemfibrozil	C_15_H_22_O_3_	250.157	15.558	73.5	Aromatic ether	
	(+)-Alantolactone	C_15_H_20_O_2_	232.146	16.028	83.9	Terpenoid	
B	Apiin	C_26_H_28_O_14_	564.1473	8.394	88.1	Flavonoids	
	Choline	C_5_H_13_NO	103.1	1.132	94.8	Cholines	
	L-(+)-Leucine	C_6_H_13_NO_2_	131.095	1.613	98.6	Amino acid	
	DL-Phenylalanine	C_9_H_11_NO_2_	165.079	2.372	98.7	Amino acid	[30]
	Citric acid	C_6_H_8_O_7_	192.026	1.394	98.4	Amino acid	[30]
	L-(+)-Valine	C_5_H_11_NO_2_	117.079	1.205	95	Carboxylic acid	
	Adenine	C_5_H_5_N_5_	135.055	1.213	99.3	Aminopurine	
	L-Proline	C_5_H_9_NO_2_	115.064	1.164	88.9	Amino acid	[31]
	DL-Malic acid	C_4_H_6_O_5_	134.021	1.297	87.4	Hydrocarboxylic acid	
	4-Oxoproline	C_5_H_7_NO_3_	129.042	1.431	97.9	Amino acid	
	L-Pyroglutamic acid	C_5_H_7_NO_3_	129.043	1.389	87.6	Amino acid	
	DL-Tryptophan	C_11_H_12_N_2_O_2_	204.09	4.97	98.1	Amino acid	
	3-Indoleacrylic acid	C_11_H_9_NO_2_	187.063	4.968	91.9	Indoles	[30]
	DL-Glutamic acid	C_5_H_9_NO_4_	147.053	1.191	95.1	Amino acid	
	Dl-Glutamine	C_5_H_10_N_2_O_3_	146.069	1.201	97.7	Amino acid	
	L-(+)-Leucine	C_6_H_13_NO_2_	131.095	1.315	85.4	Amino acid	
M	Rutin	C_27_H_30_O_16_	610.15	8.478	95.1	Flavonoids	[5,32,33]
	Isoquercetin	C_21_H_20_O_12_	464.1	8.769	92.8	Flavonol	[32]
	Vanillyl mandelic acid	C_9_H_10_O_5_	198.052	7.996	75.6	Phenolic	
	Choline	C_5_H_13_NO	103.1	1.093	99.2	Amino acid	[34]
	L-Proline	C_5_H_9_NO_2_	115.064	1.127	99.4	Amino acid	[35]
	Malic acid	C_4_H_6_O_5_	134.021	1.223	99.2	Organic acid	[34]
	Seratrodast	C_22_H_26_O_4_	354.183	18.538	97.2	Quinone derivative	
	Piclamilast	C_18_H_18_C_l2_N_2_O_3_	380.071	1.102	95.1	Benzamida	
	Gallic acid	C_7_H_6_O_5_	170.021	1.976	98.7	Phenols	[32,36]
	DL-Phenylalanine	C_9_H_11_NO_2_	165.079	2.372	98.5	Amino acid	[35]
	Citric acid	C_6_H_8_O_7_	192.026	1.21	94.4	Carboxylic acid	[32]
	4-Aminobenzoic acid	C_7_H_7_NO_2_	137.048	1.125	98.3	Aminobenzoic acid	
	L-(+)-Valine	C_5_H_11_NO_2_	117.079	1.199	85.8	Amino acid	[34]
	L-Pyroglutamic acid	C_5_H_7_NO_3_	129.043	1.196	88.9	Amino acid	
	Methylmalonic acid	C_4_H_6_O_4_	118.026	1.585	96.1	Malonates acid	
	Schisandrin	C_24_H_32_O_7_	432.214	19.508	95.4	Polycyclic	
	DL-Glutamine	C_5_H_10_N_2_O_3_	146.069	1.114	98.9	Amino acid	
	L-(+)-Leucine	C_6_H_13_NO_2_	131.095	1.553	97.9	Amino acid	[32]
	4-Oxoproline	C_5_H_7_NO_3_	129.042	1.426	98.3	Amino acid	
	Phenethylamine	C_8_H_11_N	121.089	3.597	96	Amine	
	Hex-2-ulose	C_6_H_12_O_6_	180.063	1.249	96	Carbohydrate derivative	
	Glucoheptonic acid	C_7_H_14_O_8_	226.069	1.252	96.8	Carbohydrate acid	
	Fumaric acid	C_4_H_4_O_4_	116.01	1.226	86	Fumarates acid	[32]
	DL-Tryptophan	C_11_H_12_N_2_O_2_	204.09	4.869	97.5	Amino acid	
	3-Indoleacrylic acid	C_11_H_9_NO_2_	187.063	4.87	92.8	Indoles	
	Sulfometuron methyl	C_15_H_16_N_4_O_5_S	364.083	6.847	74.4	Sulfonilurea	
	3-Phenylpropanoic acid	C_9_H_10_O_21_	150.068	18.541	93.1	Carboxylic acid	

Note: Score is match of quality.

**Table 8:** The chemical structure of the secondary metabolite in extracts S, B, and M.

Sample	Compound	Chemical Structure	Literature
S	Kaempferol	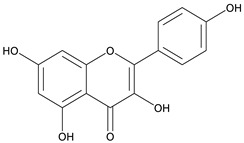	
	Retusin	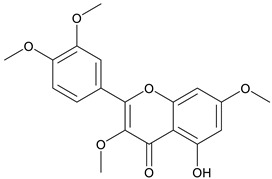	
	Isorhamnetin	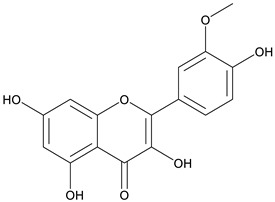	[26,27]
	Tricin (5,7,4′-trihydroxy-3′,5′-dimethoxyflavone)	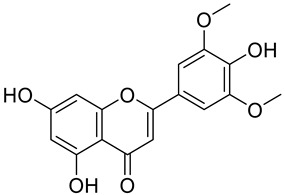	[28]
	Skrofulein	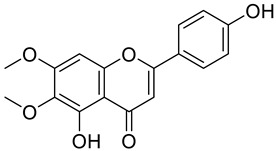	
	Hispidulin	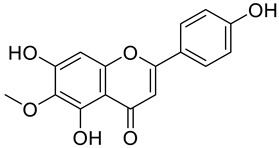	[29]
B	Apiin	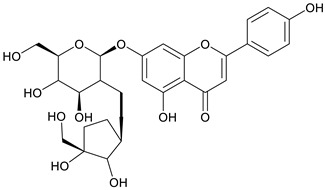	
M	Rutin	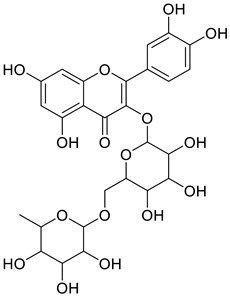	[5,32,33]
	Isoquercetin	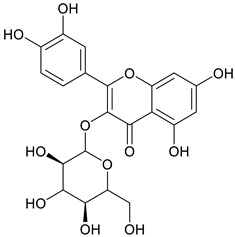	[32]

**Table 9:** Assay of TNF-α.

No	Extracts	Codes	Concentration of Cytokine (pg/mL)			% Inhibition Cytokine		
			7.8125	15.625	31.25	7.8125	15.625	31.25
1.	S (EtOH)	A		13.984	32.944		98.82	97.66
2.	S:B:M (1:0:1) (EtOH)	B	399.712	104.605	268.954	66.31	91.16	80.79
3.	S:B:M (1:1:1) (EtOH)	C	43.547	64.986	493.703	96.33	94.51	64.87
4.	Positive control (BL)	D	461.487	684.861		61.10	42.14	
5.	Positive control (tetrahidropirimidin)	E	0	0.127	14.537	100	99.99	98.97
6.	Negative control (LPS)	F	1186.39	1183.706	1405.355	0	0	0

Note: BL is BioLuric, LPS is Lipopolysaccharides.

**Table 10:** Assay of IL-6.

No	Extracts	Codes	Concentration of Cytokine (pg/mL)			% Inhibition Cytokine		
			7.8125	15.625	31.25	7.8125	15.625	31.25
1.	S (EtOH)	A		0	1.466		100	99.08
2.	S:B:M (1:0:1) (EtOH)	B	0	0	0	100	100	100
3.	S:B:M (1:1:1) (EtOH)	C	0	0	17.208	100	100	89.16
4.	Positive control (BL)	D	0	0		100	100	
5.	Positive control (tetrahidropirimidin)	E	0	0	3.418	100	100	97.85
6.	Negative control (LPS)	F	122.448	150.386	158.687	0	0	0

Note: BL is BioLuric, LPS is Lipopolysaccharides.

**Table 11:** Inhibition of TNF-α and IL-6.

No	Extracts	TNF-α			IL-6		
		7.8125	15.625	31.25	7.8125	15.625	31.25
1.	S (EtOH)		97.66	98.03		100	99.08
2.	S:B:M (1:0:1) (EtOH)	66.31	91.16	80.79	100	100	100
3.	S:B:M (1:1:1) (EtOH)	96.33	94.51	64.87	100	100	89.16
4.	Positive control (BL)	61.10	42.14		100	100	
5.	Positive control (tetrahidropirimidin)	100	99.99	98.97	100	100	97.85
6.	Negative control (LPS)	0	0	0	0	0	0

Note: BL is BioLuric, LPS is Lipopolysaccharides.

## Data Availability

The original contributions in this study are included in the article. Further inquiries can be directed to the corresponding author.
